# Assessing misophonia as a potential comorbidity in migraine patients compared to controls: a cross-sectional study

**DOI:** 10.3389/fneur.2025.1545520

**Published:** 2025-07-16

**Authors:** Aysenur Sahin, Seda Cakir, Elif Ilgaz Aydinlar, Mustafa Ertas, Pınar Yalınay Dikmen

**Affiliations:** ^1^Department of Neurology, Faculty of Medicine, Acibadem University School of Medicine, Istanbul, Türkiye; ^2^Department of Neurology, Faculty of Medicine, Istanbul University, Istanbul, Türkiye

**Keywords:** migraine, misophonia, comorbidity, misophonia assessment, migraine disability

## Abstract

**Objective:**

This study aimed to investigate the prevalence and symptom severity of misophonia among individuals with migraine, and to explore its clinical and psychological correlates.

**Background:**

Misophonia is a neurobehavioral condition characterized by intense emotional and physiological reactions to specific everyday sounds, such as chewing or tapping. Although misophonia has been associated with increased sensory sensitivity and psychiatric comorbidities, its relationship with other sensory processing disorders-particularly migraine-remains underexplored.

**Methods:**

In this cross-sectional study, 205 migraine patients and 205 healthy controls completed validated scales assessing misophonia symptoms, psychiatric comorbidities, and migraine-related disability. Statistical comparisons and univariate linear regression analyses were performed to identify predictors of misophonia.

**Results:**

Our findings revealed a significantly higher prevalence of misophonia among individuals with migraine compared to healthy controls (44.9% vs. 17.6%). Misophonia symptoms were not only more common but also more severe in the migraine group. Migraine patients with comorbid misophonia scored significantly higher on the Headache Impact Test-6 and all components of the Migraine Disability Assessment Scale compared to those without misophonia. Sensory sensitivities such as photophobia, phonophobia, osmophobia, and allodynia were also more frequent and intense among migraine patients with misophonia. Furthermore, these patients exhibited significantly higher levels of anxiety, stress, and obsessive-compulsive symptoms. Regression analyses revealed that stress, obsessive-compulsive symptoms, allodynia, and migraine-related disability were significant predictors of misophonia scores.

**Conclusion:**

Misophonia is a common and clinically significant comorbidity in migraine, associated with heightened sensory sensitivities, increased psychiatric burden, and greater functional impairment. The co-occurrence of these conditions may be underpinned by shared neurobiological mechanisms, particularly networks mediating sensory-emotional integration. Further longitudinal and neurobiological research is warranted to clarify causal relationships and inform targeted interventions.

## Introduction

Migraine and misophonia are both characterized by altered sensory processing and heightened reactivity to stimuli. Migraine affects over 1 billion people globally and has a lifetime prevalence of approximately 20% in women and 8% in men. It is the third most common disease worldwide and is recognized as one of the leading causes of disability, significantly impacting quality of life (1, 2). Migraine significantly impairs quality of life due to recurrent headaches, often accompanied by sensory symptoms such as photophobia, phonophobia, and allodynia (3). Migraine is believed to result from increased neuronal excitability and dysfunction in cortical and subcortical circuits, particularly those involving the brainstem, thalamus, anterior insula, and the trigeminovascular system (4, 5). Although pain remains the hallmark feature, migraine is now increasingly recognized as a complex neurological disorder that affects multiple domains—including sensory processing, cognition, and affective regulation (6, 7).

Misophonia, on the other hand, is a neurobehavioral condition marked by intense emotional responses to specific everyday sounds like chewing or breathing—stimuli that are otherwise considered benign (8, 9). Studies, such as those by Kumar et al., have shown that individuals with misophonia display exaggerated emotional and auditory cortical responses, suggesting abnormal sensory-emotional integration (10). These sounds can trigger intense feelings of anger, anxiety, or disgust, alongside autonomic symptoms like sweating or increased heart rate, and may even result in social withdrawal or impaired daily functioning (11–13).

Although phonophobia is a well-recognized feature of migraine, misophonia is a distinct condition with unique emotional and behavioral consequences. The neurophysiological model of misophonia posits that conditioned reflexes between the auditory pathways, limbic system, and autonomic nervous system contribute to hypersensitivity (14–16), a mechanism that closely mirrors sensory hyperreactivity seen in migraine. Moreover, both conditions share a high rate of psychiatric comorbidities such as anxiety and depression (17–20) and are associated with functional changes in brain areas like the limbic and salience networks. These shared features point toward a potential common pathophysiology, although the exact nature of their interaction remains unclear.

Despite these insights, little is known about the prevalence and impact of misophonia in migraine patients. Previous studies have not systematically assessed the comorbidity of misophonia in this population, nor its potential influence on migraine severity or associated sensory disturbances. Given the overlapping shared abnormalities in sensory processing and psychiatric comorbidities suggest that misophonia may be more prevalent in individuals with migraine. These findings support the consideration of misophonia as a comorbidity of migraine. The aim of this study is to assess the prevalence of misophonia in individuals with migraine compared to healthy controls, and to examine how misophonia influences clinical severity, psychiatric symptoms, and sensory hypersensitivity in this population.

## Methods

### Study population

**Figure 1:**
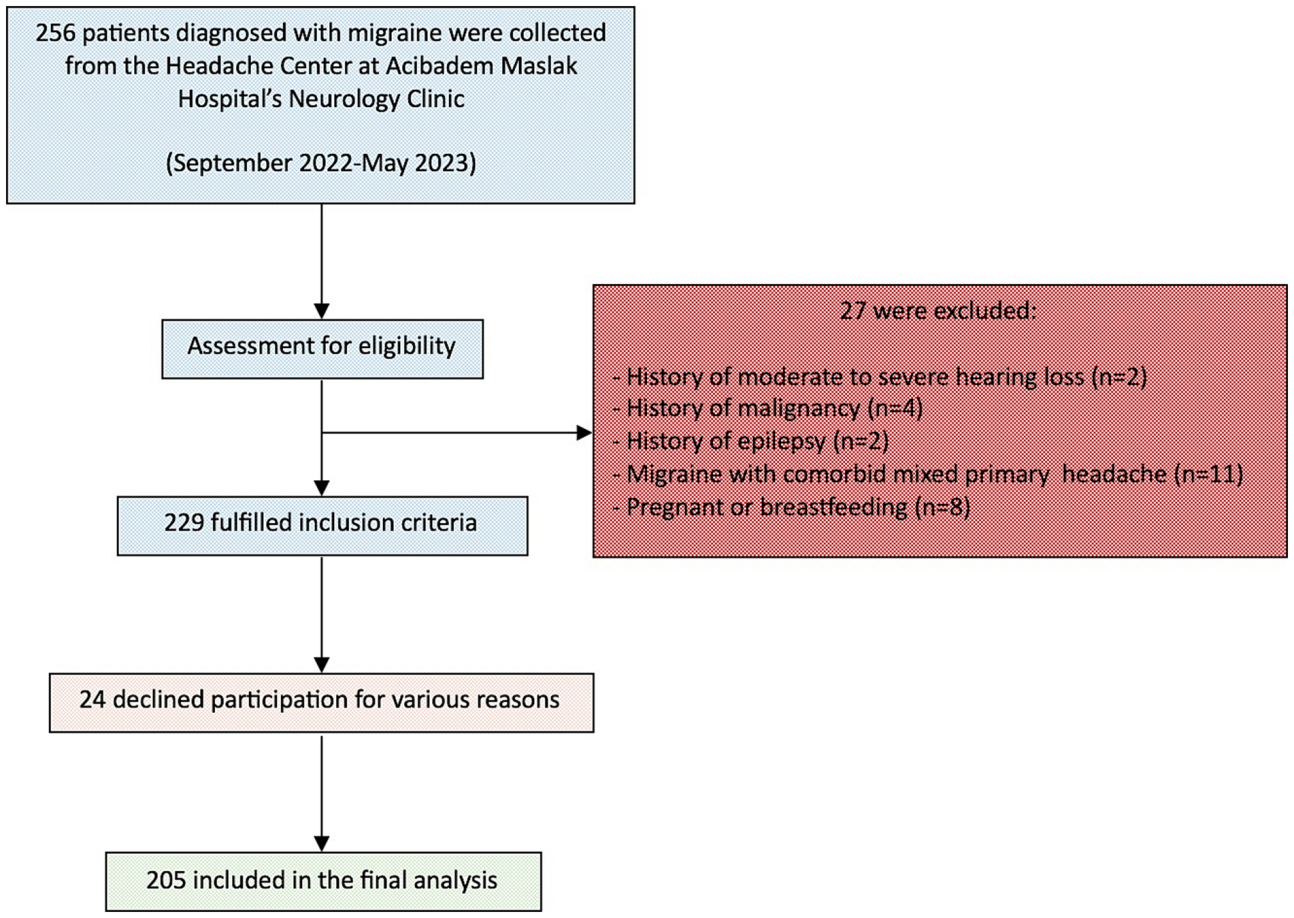
Flowchart of the participants’ enrollment process.

In this cross-sectional study, data were collected from 205 migraine patients and 205 healthy controls at Acibadem Maslak Hospital’s Neurology Clinic between September 1, 2022, and May 30, 2023. Figure 1 illustrates the participant flow throughout the study. This study was approved by the Acibadem University School of Medicine Ethics Committee (ATADEK 2022–14/29) and conducted in line with institutional guidelines and the Helsinki Declaration (2013). Written informed consent was obtained from all participants.

### Inclusion and exclusion criteria

Participants were eligible for inclusion if they were between 18 and 65 years of age, literate, capable of providing informed consent, and possessed sufficient cognitive and language abilities to complete self-report questionnaires reliably.

For the Migraine Group (MG): A diagnosis of migraine (episodic or chronic; with or without aura) confirmed by expert neurologists specializing in headache disorders (PYD, EIA) according to the International Classification of Headache Disorders, 3rd edition (ICHD-3) (21).

For the Control Group (CG): No lifetime history of migraine or frequent other primary headache disorders.

Participants in the CG were recruited from hospital personnel, administrative staff, and the non-migraine-affected relatives of patients, matched for age and sex distribution to the MG.

Exclusion criteria were history of major neurological or neurodevelopmental disorders (e.g., epilepsy, stroke, multiple sclerosis, dementia), diagnosis of any major psychiatric disorder requiring hospitalization within the past year (e.g., schizophrenia, bipolar disorder), current substance use disorder or use of psychotropic medication within the past 30 days, pregnancy or lactation at the time of the study, presence of any severe medical condition that could interfere with participation (e.g., malignancy, renal failure) and inability to understand or complete study procedures due to language barriers, cognitive impairment, or sensory deficits.

### Groups

**Figure 2:**
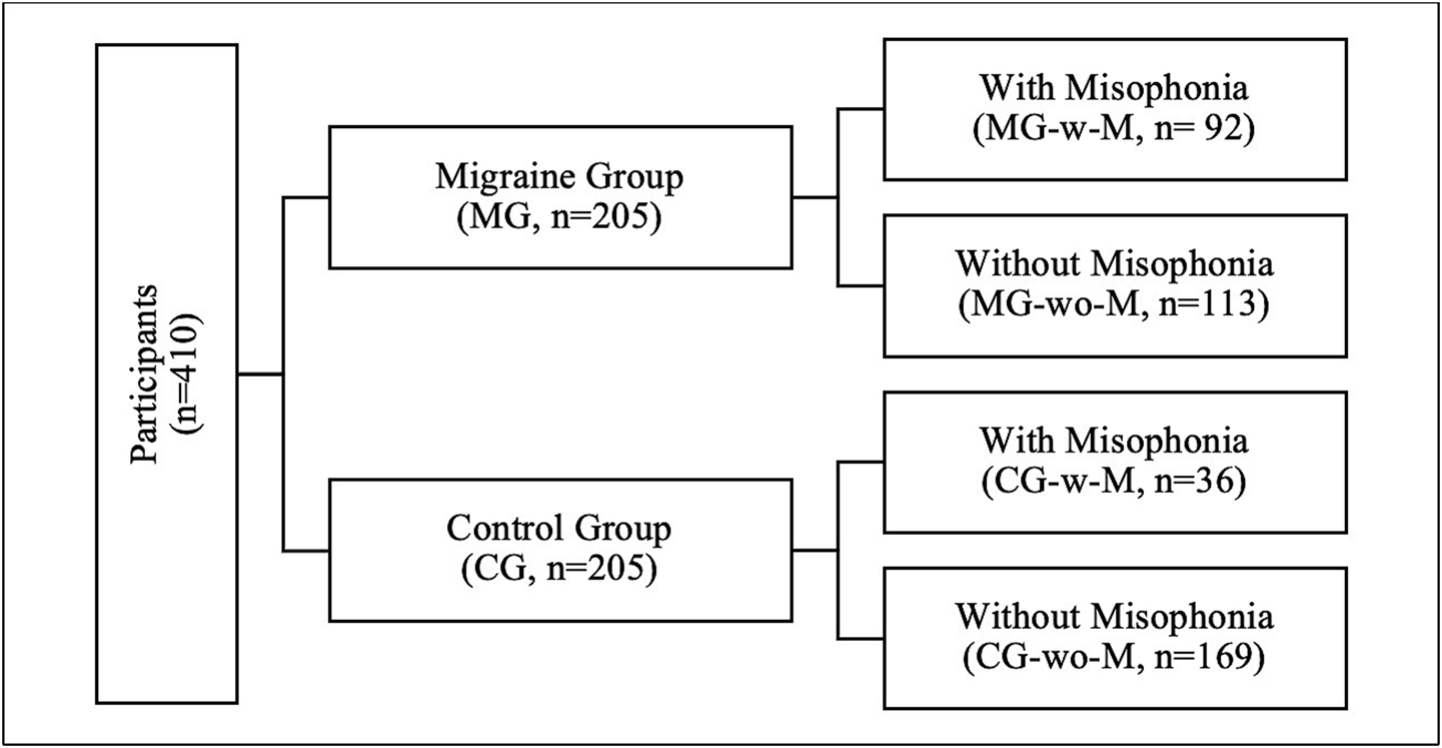
Schematic diagram: group definitions and analytic comparisons.

In this study, participants were categorized into three groups for analysis (Figure 2).

Group 1: Migraine Group (MG) vs. Control Group (CG).

Group 2: Migraine Group with Misophonia (MG-w-M) vs. Control Group with Misophonia (CG-w-M).

Group 3: Migraine Group with Misophonia (MG-w-M) vs. Migraine Group without Misophonia (MG-wo-M).

### Assessments

All participants completed a standardized battery of assessments during a 20-min face-to-face interview conducted by a single neurology resident. These assessments included the Sociodemographic Data Form (Appendix 1), the Headache Impact Test (HIT-6), the Numeric Rating Scale (NRS) for headache severity, the Misophonia Questionnaire (MQ), the Depression Anxiety Stress Scale-21 (DASS-21), and the Yale-Brown Obsessive-Compulsive Scale-Self-Report Form (Y-BOCS-SR). In addition, participants in the Migraine Group (MG) were administered three supplementary instruments specific to headache characteristics: the Clinical Features Data Form (Appendix 2), the Migraine Disability Assessment Scale (MIDAS), and the Allodynia Symptom Checklist (ASC-12).

### Sociodemographic data form

This researcher-designed form gathers data on participants’ age, gender, education level, smoking habits and alcohol consumption, exercise habits, medical history, comorbid disorders, medications used regularly, and family history with migraine.

### Clinical features data form

This form, developed by researchers following International Headache Society guidelines, includes questions regarding migraine type, age of onset, the number of headache days in the past month and 3 months, medication overuse headache (MOH), the presence and severity of ictal/interictal photophobia, phonophobia, and osmophobia, as well as current and past preventive treatments for migraine (22–24).

### Headache impact test

The HIT-6 assesses the impact of headache on social, role, and cognitive functioning, vitality, and psychological distress (25, 26). The test comprises six questions, each rated on a 5-point Likert scale (6—never, 8—rarely, 10—sometimes, 11—very often, 13—always). The total score ranges from 36 to 78, categorized as no or little impact (≤ 49 scores), some impact (50–55 scores), substantial impact (56–59 scores), and severe impact (≥ 60 scores).

### Migraine disability assessment scale

The MIDAS measures migraine-related disability over the past 3 months, focusing on productivity losses across three domains (work/school, household chores, and family/social/leisure activities) using five questions (27, 28). Disability levels are categorized as no or minimal (0–5 days), mild (6–10 days), moderate (11–20 days), and severe (> 21 days) based on the number of days lost and migraine severity.

### Allodynia symptom checklist

The ASC-12 evaluates presence and severity of cutaneous allodynia (CA) by assessing discomfort or pain on the skin during migraine attacks across 12 specific activities (e.g., combing hair, shaving) using a 6-point Likert scale (0 points-does not apply to me/never/rarely, 1 point—less than half the time, 2 points—half the time or more). The total score ranges from 0 to 24, with CA classified as never (0–2 points), mild (3–5 points), moderate (6–8 points), and severe (≥ 9 points) (29, 30).

### Misophonia questionnaire

The MQ evaluates sensitivity to specific sounds and the associated emotional and behavioral responses that are characteristic of misophonia (13, 31). The original questionnaire consists of three sections. The first part, named the “*Misophonia Symptom Scale*,” includes seven items querying sensitivity to everyday sounds (e.g., nasal sounds, rustling). The second part, titled the “*Misophonia Emotions and Behaviors Scale,*” consists of 10 statements assessing emotions responses (e.g., anxious/ distressed) and behavioral reactions (e.g., verbally aggressive) to triggers. The first two parts rated on a 5-point Likert scale (0—not at all true, 1—rarely true, 2—sometimes true, 3—often true, 4—always true).

The total score from these 17 statements ranges from 0 to 68, with higher scores indicating greater symptom severity. The last part of the questionnaire, the “*Misophonia Severity Scale*,” was adapted from the National Institute of Mental Health Global Obsessive-Compulsive Scale (1982), asks participants to rate the severity of trigger sounds, and their impact on daily life on a scale from 1 to 15. A score of ≥ 7 indicates clinically significant misophonia, with at least moderate sound sensitivity.

In the Turkish validation and reliability study, exploratory and confirmatory factor analyses revealed that the ‘Misophonia Emotions and Behaviors’ part divides into two distinct factors: ‘Avoidance and Internalization’ and ‘Aggression and Externalization’ (31). In this study, the MQ was applied following this revised structure.

The Misophonia Questionnaire (MQ) was used to determine whether participants had misophonia. Those with a score of ≥7 on the Misophonia Severity Scale were classified as having clinically significant misophonia and were included in the migraine group with misophonia (MG-w-M) or the control group with misophonia (CG-w-M), respectively.

### Depression anxiety stress scale

This scale consists of 21 statements, assessing symptoms of depression, anxiety, and stress with seven items for each domain (32, 33). Participants rate each statement over the past week on a 4-point Likert scale (0—did not apply to me at all, 1—applied to me to some degree, 2—applied to me to a considerable degree, 3—applied to me very much or most of the time).

The total scores for each domain range from 0 to 21, with scores above specific thresholds indicating potential issues: for depression, normal (0–4 points), mild (5–6 points), moderate (7–10 points), severe (11–13 points), extremely severe (≥ 14 points); for anxiety, normal (0–3 points), mild (4–5 points), moderate (6–7 points), severe (8–9 points), extremely severe (≥ 10 points); for stress, normal (0–7 points), mild (8–9 points), moderate (10–12 points), severe (13–16 points), extremely severe (≥ 17 points).

### Yale-Brown obsession compulsion scale—self-report form

The Y-BOCS-SR begins by defining obsessions and compulsions, accompanied by examples (34, 35). Participants are then asked to rate five obsessions, and five compulsions experienced over the past week, using a 5-point Likert scale (0—none, 1—mild, 2—moderate, 3—severe, 4—extreme). The scale evaluates the time spent on obsessions and compulsions, their interference in daily life, and the level of resistance. The total score ranges from 0 to 40, categorized clinically as no clinical level (0–7 points), mild (8–15 points), moderate (16–23 points), severe (21–31 points), extremely severe (32–40 points).

### Statistical analysis

Based on a misophonia prevalence of 12.8% in Turkey, a sample size was calculated via computer software (Clincalc) and revealed at least 200 individuals to be included in the study with type 1 error (α = 0.05) and a 95% confidence interval (35, 36). The final sample included 205 migraine patients and 205 matched healthy controls. This analysis represents the primary evaluation of the dataset. The final dataset was complete, with no missing data.

Data distribution was evaluated using the Shapiro–Wilk test. Continuous variables are presented as means and standard deviations (SD) or medians with ranges where appropriate. Categorical variables are reported as frequencies and percentages (n, %). Comparisons between categorical variables were performed using the Chi-square (χ^2^) test. Continuous variables were analyzed using independent samples *t*-tests.

**Figure 3:**
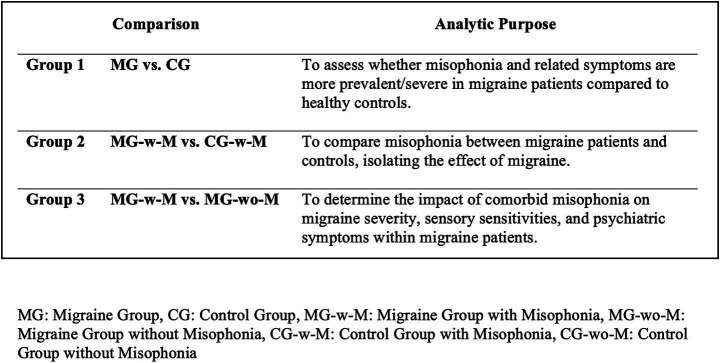
Schematic diagram: analytic comparisons.

Group comparisons were conducted across predefined analytic frameworks, as outlined in Figure 3.

The MG versus the CG to assess group-level differences in misophonia prevenance and symptom severity.

The MG-w-M versus the CG-w-M to isolate the effect of migraine on misophonia symptoms.

The MG-w-M versus the MG-wo-M to explore the clinical and psychiatric impact of comorbid misophonia among individuals with migraine.

**Figure 4:**
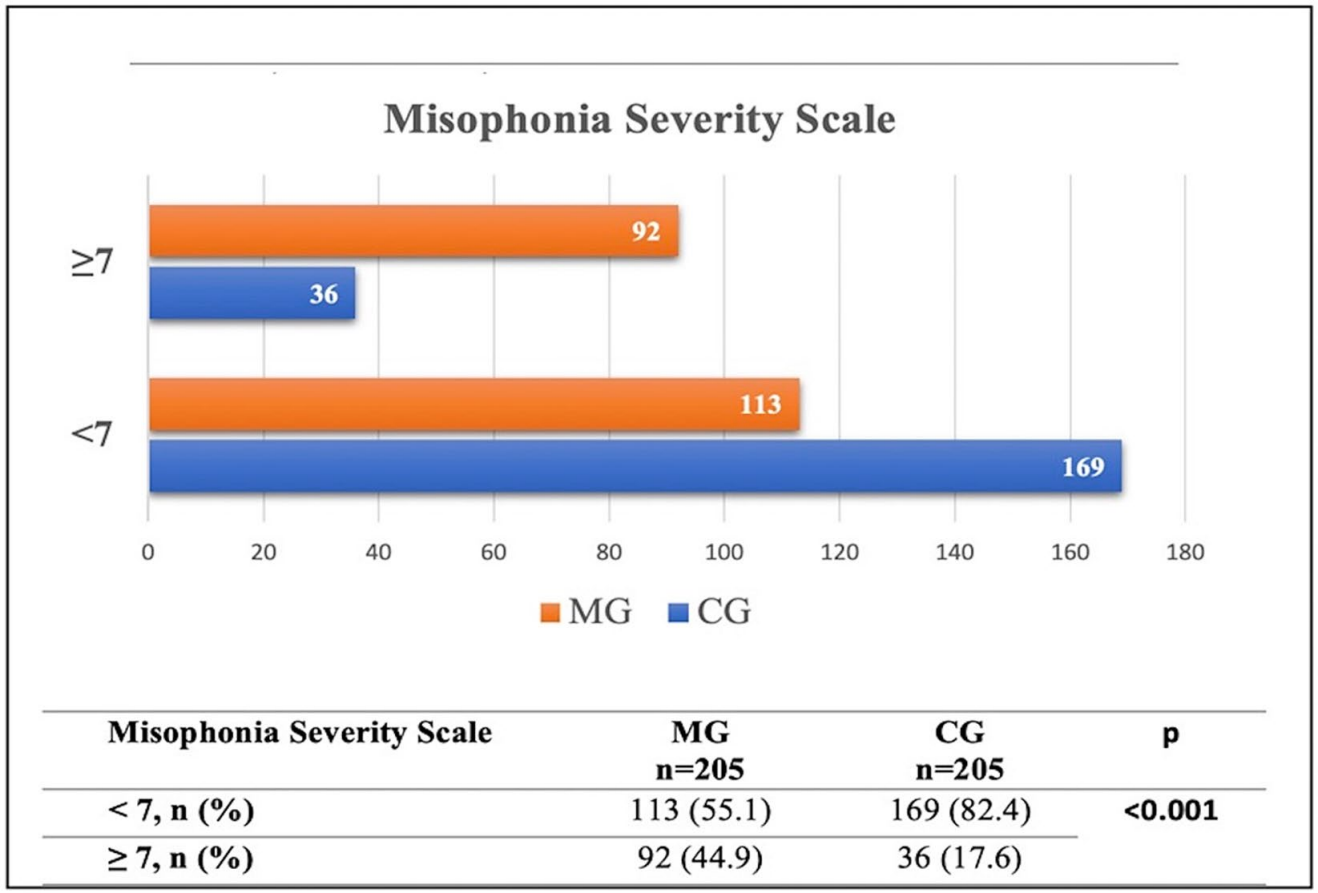
Comparison of misophonia severity scale cut-off values between the MG and CG groups.

To identify predictors of misophonia severity, univariate linear regression analyses were conducted using the total Misophonia Questionnaire score as the dependent variable. Each clinical, sensory, and psychiatric variable was entered separately as an independent predictor. Results are reported with unstandardized (B) and standardized (β) coefficients, standard errors (SE), *t*-values, *p*-values, and coefficients of determination (R^2^). Data were analyzed using SPSS version 26.0 (IBM Corp., Armonk, NY). All tests were two-tailed, and a *p*-value of <0.05 was considered statistically significant (Figure 4).

## Results

### Sociodemographic features

A total of 205 migraine patients (MG) and 205 healthy controls (CG) were included in the analyses. Sociodemographic characteristics were comparable across groups in terms of age and sex. However, the MG had significantly higher rates of family history of migraine, as well as previous diagnoses of depression and anxiety (Table 1).

**Table 1:** Demographic characteristics of the groups.

Variables	Group 1			Group 2			Group 3		
	MG *n* = 205	CG *n* = 205	*p*	MG-w-M *n* = 92	CG-w-M *n* = 36	*p*	MG-w-M *n* = 92	MG-wo-M *n* = 113	*p*
Female sex ^a^, *n* (%)	173 (84.4)	173 (84.4)	χ^2^(1) = 0.0 *p* = 1	85 (92.4)	34 (94.4)	χ^2^(1) = 0.167 *p* = 0.683	85 (92.4)	88 (77.9)	χ^2^(1) = 8.1 ***p* = 0.004**
Age (years) ^b^, (Mean, SD) 95% CI	36.5 (9.4)	36.5 (10.0)	*p* = 0.956 [−2,1.8]	36.5 (9.0)	36.7 (10.4)	*p* = 0.911 [−4, 3.5]	36.5 (9.3)	36.4 (9.6)	*p* = 0.927 [−2.8, 2.5]
Family history of migraine^a^, *n* (%)	131 (63.9)	41 (20.0)	χ^2^(1) = 81.1 ***p* < 0.001**	61 (66.3)	6 (16.7)	χ^2^(1) = 25.6 ***p* < 0.001**	31 (33.7)	43 (38.1)	χ^2^(1) = 0.417 *p* = 0.518
History of depression ^a^, *n* (%)	33 (16.1)	7 (3.4)	χ^2^(1) = 18.7 ***p* < 0.001**	18 (19.6)	1 (2.8)	χ^2^(1) = 5.8 ***p* = 0.016**	18 (19.6)	15 (13.3)	χ^2^(1) = 1.46 *p* = 0.223
History of anxiety ^a^, *n* (%)	25 (12.2)	8 (3.9)	χ^2^(1) = 9.5 ***p* = 0.002**	16 (17.4)	1 (2.8)	χ^2^(1) = 4.8 ***p* = 0.028**	16 (17.4)	9 (8.08)	χ^2^(1) = 4.28 ***p* = 0.040**

*n*: Number of participants, MG: Migraine Group, CG: Control Group, MG-w-M: Migraine Group with Misophonia, CG-w-M: Control Group with Misophonia, MG-wo-M: Migraine Group without Misophonia, SD: Standard Deviation, CI: Confidence Interval, (df): Degrees of freedom, *p* < 0.05.

a Chi-square (χ^2^) test.

b Independent samples *t*-test.

Statistically significant values are indicated in bold.

### Clinical features of MG

In our cohort, 77.6% of the patients experienced episodic migraine frequency, and 67.8% were using preventive treatment (Table 2). The presence of aura was notably higher in MG-w-M (37%) compared to MG-wo-M (16.8%) [χ2(1) = 10.7, *p* = 0.001]. The mean number of headache days over the last month for MG was 8.6 (6.7), with a mean MIDAS total score of 19.9 (21.5). Additionally, 58.1% of the patients experienced moderate or severe disability due to migraine.

**Table 2:** Clinical characteristics of migraine patients in the Groups.

Migraine History	MG *n* = 205	MG-w-M *n* = 92	MG-wo-M *n* = 113	*p*
Diagnosis ^a^, *n* (%)				
EM	159 (77.6)	67 (72.8)	92 (81.4)	χ^2^(1) = 2.1 *p* = 0.143
CM	46 (22.4)	25 (27.2)	21 (18.6)	
Presence of aura	53 (25.8)	34 (37.0)	19 (16.8)	χ^2^(1) = 10.7 ***p* = 0.001**
Patients with MOH ^a^, *n* (%)	29 (63.0)	28 (30.4)	25 (22.1)	χ^2^(1) = 1.8 *p* = 0.176
Patients on preventive migraine treatment ^a^, *n* (%)	139 (67.8)	65 (70.7)	74 (65.5)	χ^2^(1) = 0.6 *p* = 0.431
	Mean (SD)	Mean (SD)		
Age at migraine onset ^b^	22.3 (8.7)	22.38 (9.2)	22.31 (8.4)	95% CI [−2.5, 2.4] *p* = 0.954
Duration of migraine ^b^ (years)	14.1 (9.9)	14.20 (9.9)	14.04 (10.0)	95% CI [−2.9, 2.6] *p* = 0.160
Headache days in last month ^b^	8.6 (6.7)	9.46 (7.2)	7.89 (6.2)	95% CI [−3.4, 0.3] *p* = 0.099
MIDAS-A ^b^	23.1 (18.7)	26.13 (19.3)	20.74 (18.0)	95% CI [−10.5, 0.2] ***p* = 0.041**
MIDAS-B ^b^	7.2 (1.5)	7.50 (1.5)	7.07 (1.4)	95% CI [−0.8, 0.01] ***p* = 0.045**
MIDAS total score ^b^	19.9 (21.5)	25.47 (26.8)	15.39 (14.6)	95% CI [−15.9, 4.3] ***p* = 0.001**
MIDAS grade n ^a^, (%)				
1 (No or minimal)	42 (20.5)	12 (13.0)	30 (26.5)	χ^2^(3) = 11.7 ***p* = 0.008**
2 (Mild)	44 (21.5)	16 (17.4)	28 (24.8)	
3 (Moderate)	50 (24.4)	23 (25.0)	27 (23.9)	
4 (Severe)	69 (33.7)	41 (44.6)	28 (24.8)	

n: Number of participants, MG: Migraine Group, MG-w-M: Migraine Group with Misophonia, MG-wo-M: Migraine Group without Misophonia, EM: Episodic Migraine, CM: Chronic Migraine, MOH: Medication overuse headache, MIDAS: Migraine Disability Assessment Test, MIDAS-A: On how many days in the last 3 months did you have a headache?, MIDAS-B: On a scale of 0–10, on average how painful were these headaches?, CI: Confidence Interval, (df): Degrees of freedom, SD: Standard Deviation, *p* < 0.05.

a Chi-square (χ^2^) test.

b Independent samples *t*-test.

Statistically significant values are indicated in bold.

In clinical comparisons, migraine patients with comorbid misophonia (MG-w-M) reported significantly greater disability, as reflected in higher MIDAS total scores (*p* = 0.001) and MIDAS-A scores (*p* = 0.041) than migraine patients without misophonia (MG-wo-M). Nearly half of MG-w-M participants were categorized in the “severe disability” grade (44.6%), compared to 24.8% in the MG-wo-M group (Table 2).

### Comparison of sensory features and allodynia scores

Photophobia, phonophobia, and osmophobia were evaluated based on symptom severity, classified as none, mild, moderate, or severe, in both migraine patients and healthy controls. Table 3 presents the severity distributions of ictal and interictal photophobia, phonophobia, and osmophobia, along with ASC-12 scores specifically within the MG.

**Table 3:** Comparison of sensory features and allodynia scores by groups.

GROUPS	Interictal Photophobia *n* (%)			Ictal Photophobia *n* (%)			Interictal Phonophobia *n* (%)			Ictal Phonophobia *n* (%)			Interictal Osmophobia *n* (%)			Ictal Osmophobia *n* (%)			ASC-12 scores *n* (%)		
Severity^*^	Mi	Mo	Se	Mi	Mo	Se	Mi	Mo	Se	Mi	Mo	Se	Mi	Mo	Se	Mi	Mo	Se	Mi	Mo	Se
MG *n* = 205	62 (30.2)	40 (19.5)	8 (3.9)	18 (8.8)	53 (25.9)	115 (56.1)	78 (38.0)	44 (21.5)	16 (7.8)	16 (7.8)	64 (31.2)	111 (54.1)	29 (14.1)	48 (23.4)	25 (12.2)	22 (10.7)	40 (19.5)	91 (44.4)	59 (28.8)	46 (22.4)	30 (14.7)
MG-w-M *n* = 92	35 (38.0)	22 (23.9)	4 (4.3)	3 (3.3)	15 (16.3)	69 (75.0)	33 (35.9)	29 (31.5)	10 (10.9)	4 (4.3)	20 (21.7)	65 (70.7)	16 (17.4)	28 (30.4)	20 (21.7)	7 (7.6)	16 (17.4)	56 (60.9)	25 (27.2)	28 (30.4)	22 (23.9)
MG-wo-M *n* = 113	27 (23.9)	18 (15.9)	4 (3.5)	15 (13.3)	38 (33.6)	46 (40.7)	45 (39.8)	15 (13.3)	6 (5.3)	12 (10.6)	44 (38.9)	46 (40.7)	13 (11.5)	20 (17.7)	5 (4.4)	15 (13.3)	24 (21.2)	35 (31.0)	34 (30.1)	18 (15.9)	8 (7.1)
*p*	χ^2^(3) = 10.9 ***p* = 0.013**			χ^2^(3) = 25.0 ***p* < 0.001**			χ^2^(3) = 16.2 ***p* = 0.001**			χ^2^(3) = 18.9 ***p* < 0.001**			χ^2^(3) = 30.3 ***p* < 0.001**			χ^2^(3) = 20.4 ***p* < 0.001**			χ^2^(3) = 26.7 ***p* < 0.001**		

* Severity: Mi: Mild, Mo. Moderate, Se: Severe.

*N*: Number of participants, MG: Migraine Group, MG-w-M: Migraine Group with Misophonia, CG-w-M: Control Group with Misophonia, MG-wo-M: Migraine Group without Misophonia, ASC-12: Allodynia Symptom Checklist, (df): Degree of freedom, *p* < 0.05. Participants who did not report sensory symptoms or allodynia are not presented in the table. Chi-square (χ^2^) test used for comparison of the groups. Statistically significant values are indicated in bold.

### Group 1: MG vs. CG sensory symptoms

Among migraine patients, interictal photophobia was reported as mild in 62 individuals (30.2%), moderate in 40 (19.5%), and severe in 8 (3.9%). In contrast, healthy controls reported photophobia as mild in 64 individuals (31.2%), moderate in 13 (6.3%), and none at the severe level. For interictal phonophobia, migraine patients reported mild symptoms in 78 individuals (38.0%), moderate in 44 (21.5%), and severe in 16 (7.8%). In the CG, phonophobia was reported as mild in 54 individuals (26.3%), moderate in 15 (7.3%), and severe in 6 (2.9%). Similarly, interictal osmophobia was reported as mild by 29 migraine patients (14.1%), moderate by 48 (23.4%), and severe by 25 (12.2%). Among healthy controls, osmophobia was reported as mild by 52 individuals (25.4%), moderate by 17 (8.3%), and severe by 15 (7.3%). Chi-square analyses showed that the severity distributions of all three sensory disturbances were significantly higher in the MG compared to controls (photophobia: χ^2^(3) = 26.7, *p* < 0.001; phonophobia: χ^2^(3) = 43.3, *p* < 0.001; osmophobia: χ^2^(3) = 25.3, *p* < 0.001).

### Group 2: MG-w-M vs. CG-w-M sensory symptoms

In Group 2, interictal photophobia was reported as mild by 35 migraine patients with misophonia (38.0%), moderate by 22 (23.9%), and severe by 4 (4.3%). In contrast, among controls with misophonia, photophobia was reported as mild by 10 individuals (27.8%), moderate by 5 (13.9%), and none reported severe symptoms. Regarding interictal phonophobia, 33 MG-w-M participants (35.9%) reported mild, 29 (31.5%) moderate, and 10 (10.9%) severe symptoms. In the CG-w-M group, 12 individuals (33.3%) reported mild phonophobia, 12 (33.3%) moderate, and 5 (13.9%) severe symptoms. For interictal osmophobia, 16 migraine patients (17.4%) reported mild, 28 (30.4%) moderate, and 20 (21.7%) severe symptoms. Among control participants, osmophobia was reported as mild by 10 individuals (27.8%), moderate by 5 (13.9%), and severe by 6 (16.7%). Statistical comparisons showed no significant differences in severity distribution of these symptoms between the groups.

### Group 3: MG-w-M vs. MG-wo-M sensory symptoms

In Group 3, comparisons between migraine patients with misophonia (MG-w-M) and those without misophonia (MG-wo-M) revealed statistically significant differences in the severity of all evaluated sensory symptoms. Significant group differences were found for interictal photophobia [χ^2^(3) = 10.9, *p* = 0.013], ictal photophobia [χ^2^(3) = 25.0, *p* < 0.001], interictal phonophobia [χ^2^(3) = 16.2, *p* = 0.001], ictal phonophobia [χ^2^(3) = 18.9, *p* < 0.001], interictal osmophobia [χ^2^(3) = 30.3, *p* < 0.001], and ictal osmophobia [χ^2^(3) = 20.4, *p* < 0.001], indicating a higher burden of sensory hypersensitivities among migraine patients with comorbid misophonia (Table 3).

### Clinical scales comparisons between groups

In MG, a total of 34.1% of migraine patients (*n* = 70) reported no allodynia. However, 59 individuals (28.8%) had mild allodynia, 46 (22.4%) had moderate, and 30 (14.7%) had severe symptoms. These results indicate that approximately 66% of migraine patients experienced at least mild allodynia, with a smaller subset (14.7%) reporting symptoms in the severe range, highlighting the clinical relevance of sensory hypersensitivity in this population (Table 3).

In Group 3, a comparison of ASC-12 scores revealed a markedly higher severity of cutaneous allodynia in migraine patients with misophonia (MG-w-M) compared to those without misophonia (MG-wo-M). In the MG-w-M group (*n* = 92), 25 individuals (27.2%) had mild allodynia, 28 (30.4%) moderate, and 22 (23.9%) severe symptoms. In contrast, among MG-wo-M patients (*n* = 113), 34 (30.1%) reported mild symptoms, 18 (15.9%) moderate, and only 8 (7.1%) severe allodynia. The distribution of ASC-12 scores differed significantly between the groups [χ^2^(3) = 26.7, *p* < 0.001], indicating a greater burden of allodynia in patients with comorbid misophonia.

Psychiatric symptom scores were significantly higher in the MG compared to the CG across all DASS-21 and Y-BOCS-SR subscales. Compared to CG-w-M, the MG-w-M group had significantly higher scores on HIT-6, NRS, and total stress scores (DASS-21). When comparing MG-w-M to MG-wo-M, the misophonia group showed significantly elevated scores across all clinical and psychiatric scales, except for headache severity (NRS), which remained similar between groups (Table 4)

**Table 4:** Comparison of clinical scales between the groups.

Variables	Group 1				Group 2				Group 3			
	MG *n* = 205 Mean (SD)	CG *n* = 205 Mean (SD)	95% CI	*p*	MG-w-M *n* = 92 Mean (SD)	CG-w-M *n* = 36 Mean (SD)	95% CI	*p*	MG-w-M *n* = 92 Mean (SD)	MG-wo-M *n* = 113 Mean (SD)	95% CI	*p*
HIT-6	65.22 (6.42)	42.90 (6.06)	[−21.1, 23.5]	***p* < 0.001**	67.13 (6.25)	44.06 (6.06)	[20.5, 25.6]	***p* < 0.001**	67.13 (6.25)	63.66 (6.16)	[−5.2, −1.7]	***p* < 0.001**
NRS	7.26 (1.52)	3.11 (1.41)	[−3.9, 4.4]	***p* < 0.001**	7.50 (1.54)	3.39 (1.15)	[3.5, 4.7]	***p* < 0.001**	7.50 (1.54)	7.07 (1.48)	[−0.8, −0.01]	***p* = 0.045**
Depression Total Score (DASS-21)	5.20 (4.66)	3.34 (3.50)	[1.1, 2.7]	***p* < 0.001**	6.79 (5.18)	4.39 (3.79)	[0.5, 4.3]	***p* = 0.013**	6.79 (5.18)	3.90 (3.73)	[−4.1, −1.7]	***p* < 0.001**
Anxiety Total Score (DASS-21)	4.63 (3.91)	2.95 (2.82)	[1, 2.3]	***p* < 0.001**	6.04 (4.45)	3.97 (3.22)	[0.5, 3.7]	***p* = 0.012**	6.04 (4.45)	3.48 (2.97)	[−3.6, −1.5]	***p* < 0.001**
Stress Total Score (DASS-21)	7.76 (4.44)	5.41 (3.52)	[1.6, 3.2]	***p* < 0.001**	9.93 (4.61)	7.47 (3.37)	[0.8, 4.1]	***p* = 0.004**	9.93 (4.61)	5.98 (3.40)	[−5.1, −2.8]	***p* < 0.001**
Obsession Total Score (Y-BOCS-SR)	6.26 (4.88)	5.13 (4.57)	[0.2, 2]	***p* = 0.016**	7.86 (5.05)	8.19 (4.99)	[−2.3, 1.6]	*p* = 0.735	7.86 (5.05)	4.96 (4.34)	[−4.2, −1.6]	***p* < 0.001**
Compulsion Total Score (Y-BOCS-SR)	5.04 (4.53)	4.16 (4.02)	[0.05, 1.7]	***p* = 0.038**	6.25 (4.89)	6.86 (4.74)	[−2.5, 1.3]	*p* = 0.523	6.25 (4.89)	4.05 (3.98)	[−3.4, −1]	***p* < 0.001**
Obsession and Compulsion Total Score (Y-BOCS-SR)	11.29 (9.06)	9.36 (8.22)	[0.3, 3.6]	***p* = 0.024**	14.11 (9.52)	15.06 (9.43)	[−4.6, 2.7]	*p* = 0.613	14.11 (9.52)	9.00 (8.01)	[−7.5, −2.7]	***p* < 0.001**

*n*: Number of participants, MG: Migraine Group, CG: Control Group, MG-w-M: Migraine Group with Misophonia, CG-w-M: Control Group with Misophonia, MG-wo-M: Migraine Group without Misophonia, HIT-6: Headache Impact Test, NRS: Numeric Rating Scale, DASS-21: Depression Anxiety Stress Scale-21, Y-BOCS-SR: Yale-Brown Obsession and Compulsion Scale-Self Reported Form, CI: Confidence Interval, *p* < 0.05. Independent samples *t*-test was used comparison of the groups. Statistically significant values are indicated in bold.

### Misophonia questionnaire scores of the groups

Misophonia symptoms and severity were evaluated using the Misophonia Questionnaire (MQ), including subscales for symptom frequency, emotional and behavioral responses, and overall severity. In alignment with the diagnostic framework, subscale comparisons were restricted to participants who met misophonia criteria—specifically, MG-w-M and CG-w-M. This ensured clinical interpretability and avoided misapplication of misophonia-specific metrics in individuals without the disorder.

Figure 4 presents the distribution of participants with misophonia in both the MG and CG groups. For the Misophonia Symptom Scale, the mean score in the MG was 16.52 (SD = 7.17), while in the CG it was 11.73 (SD = 6.70). Regarding the Misophonia Emotions and Behaviors Avoidance and Internalization subscale, the MG demonstrated a mean score of 12.39 (SD = 6.28), compared to 6.71 (SD = 5.81) in the CG. The mean score for Misophonia Emotions and Behaviors Aggression and Externalization was 5.85 (SD = 4.20) in the MG and 2.93 (SD = 2.96) in the CG. The total Misophonia Score was 34.73 (SD = 15.13) in the MG and 21.37 (SD = 13.66) in the CG. Finally, the Misophonia Severity Scale mean was 6.68 (SD = 3.17) in the MG and 4.10 (SD = 2.52) in the CG.

In Group 2, patients with migraine and comorbid misophonia (*n* = 92) also showed significantly higher total scores and severity scores compared to controls with misophonia (*n* = 36), with *p*-values ranging from 0.002 to 0.004. However, individual subscale differences were not statistically significant for some subcomponents (Table 5).

**Table 5:** Comparison of the misophonia questionnaire between the MG-w-M and the CG-w-M.

Variables	MG-w-M *n* = 92 Mean (SD)	CG-w-M *n* = 36 Mean (SD)	95% CI	*p*
Misophonia Symptom Scale	22.0 (3.93)	20.69 (4.34)	[−0.3, 2.9]	*p* = 0.104
Misophonia Emotions and Behaviors Avoidance and Internalization	17.12 (4.28)	16.06 (4.32)	[−0.6, 2.7]	*p* = 0.210
Misophonia Emotions and Behaviors Aggression and Externalization	8.55 (3.53)	6.47 (3.24)	[0.7, 3.4]	***p* = 0.003**
Misophonia Total Score	47.68 (7.54)	43.19 (8.10)	[01.5, 7.5]	***p* = 0.004**
Misophonia Severity Scale	9.49 (2.29)	8.19 (1.30)	[0.5, 2.1]	***p* = 0.002**

*N*: Number of participants, MG-w-M: Migraine Group with Misophonia, CG-w-M: Control Group with Misophonia, SD: Standard Deviation, CI: Confidence Interval, *p* < 0.05, Independent samples *t*-test used for comparison of the groups. Statistically significant values are indicated in bold.

Item-level analysis revealed that MG-w-M participants were more likely than CG-w-M to leave the environment in response to trigger sounds (*p* = 0.006), and to experience violent thoughts (*p* = 0.006) and anger (*p* = 0.007) in the face of such stimuli. However, perceived discomfort toward specific sound categories did not differ significantly between these groups (Table 6).

**Table 6:** Comparison of misophonia questionnaire subcomponents between migraine and control group participants with misophonia.

MQ Item	MG-w-M *n* = 92	CG-w-M *n* = 36	95% CI	*p*
Misophonia Symptom Scale, mean (SD)				
People eating	3.50 (0.87)	3.61 (0.80)	[−0.4, 0.2]	*p* < 0.509
Repetitive tapping	3.65 (0.60)	3.56 (0.69)	[−0.1, 0.3]	*p* < 0.436
Rustling	3.27 (0.90)	3.19 (0.78)	[−0.3, 0.4]	*p* = 0.653
Nasal sounds	3.26 (0.93)	3.03 (1.08)	[−0.1, 0.6]	*p* = 0.228
Throat sounds	3.16 (0.96)	2.97 (1.23)	[−0.2, 0.6]	*p* = 0.355
Consonants/vowels	1.96 (1.47)	1.44 (1.42)	[−0.06, 1]	*p* = 0.077
Environmental sounds	3.23 (1.04)	2.83 (1.13)	[−0.02, 0.8]	*p* = 0.064
Misophonia Emotions and Behaviours Scale—Avoidance and Internalization, mean (SD)				
Leave environment	3.41 (0.77)	2.94 (1.01)	[0.1, 0.8]	***p* = 0.006**
Avoid	3.17 (0.92)	3.11 (0.85)	[−0.3, 0.4]	*p* = 0.724
Cover ears	2.25 (1.52)	2.25 (1.42)	[−0.6, 0.6]	*p* = 1.0
Anxious/distressed	2.68 (1.22)	2.58 (1.15)	[−0.4, 0.6]	*p* = 0.669
Sad/depressed	1.97 (1.48)	1.75 (1.36)	[−0.4, 0.8]	*p* = 0.448
Annoyed	3.73 (0.49)	3.61 (0.59)	[−0.1, 0.3]	*p* = 0.259
Misophonia Emotions and Behaviours Scale—Aggression and Externalization, mean (SD)				
Violent thoughts	2.12 (1.47)	1.31 (1.45)	[−0.2, 1.4]	***p* = 0.006**
Angry	3.27 (0.93)	2.75 (1.05)	[0.1, 0.9]	***p* = 0.007**
Physically aggressive	0.89 (1.20)	0.53 (0.94)	[−0.1, 0.8]	*p* = 0.107
Verbally aggressive	2.11 (1.27)	1.89 (1.21)	[−0.3, 0.7]	*p* = 0.1368

*N*: Number of participants, MG-w-M: Migraine Group with Misophonia, CG-w-M: Control Group with Misophonia, SD = Standard Deviation, CI: Confidence Interval, *p* < 0.05. Independent samples *t*-test was used to comparison of the groups. Statistically significant values are indicated in bold.

### Linear regression analyses predicting Misophonia scores

Univariate linear regression analyses identified several clinical and psychiatric variables as significant predictors of total misophonia total scores. The strongest predictors included stress levels (β = 0.516, R^2^ = 0.266), obsessive-compulsive symptoms (β = 0.407, R^2^ = 0.166), and allodynia scores (β = 0.373, R^2^ = 0.139). Among migraine-related variables, both HIT-6 (β = 0.242, R^2^ = 0.059) and MIDAS scores (β = 0.202, R^2^ = 0.041) were statistically significant. Subscale-level regression revealed very strong associations between misophonia total scores and the Avoidance/Internalization (R^2^ = 0.803), Aggression/Externalization (R^2^ = 0.537), and Misophonia Severity subscales (R^2^ = 0.860), indicating the critical role of affective and behavioral reactivity in overall misophonia symptomatology (Table 7).

**Table 7:** Linear regression analyses predicting misophonia total scores.

Predictor Variable	B	SE	β	*t*	*p*-value	R^2^
Age	−0.062	0.112	−0.039	−0.551	0.582	0.001
Age at Migraine Onset	0.040	0.121	0.023	0.329	0.742	0.001
Migraine Duration (years)	−0.074	0.107	−0.048	−0.691	0.490	0.002
Headache Days in Last Month	0.344	0.156	0.153	2.208	0.028*	0.023
HIT-6 Score	0.570	0.160	0.242	3.552	<0.001**	0.059
MIDAS Total Score	0.142	0.048	0.202	2.944	0.004**	0.041
Depression Total Score (DASS-21)	1.360	0.207	0.419	6.568	<0.001**	0.175
Anxiety Total Score (DASS-21)	1.561	0.248	0.404	6.292	<0.001**	0.163
Stress Total Score (DASS-21)	1.758	0.205	0.516	8.585	<0.001**	0.266
Allodynia Score	1.564	0.273	0.373	5.724	<0.001**	0.139
Obsession Score (Y-BOCS-SR)	1.218	0.200	0.393	6.096	<0.001**	0.155
Compulsion Score (Y-BOCS-SR)	1.298	0.216	0.389	6.020	<0.001**	0.151
Y-BOCS-SR Total Score	0.680	0.107	0.407	6.355	<0.001**	0.166
Misophonia Symptom Scale	1.866	0.069	0.885	27.055	<0.001**	0.783
Avoidance/Internalization Subscale	2.159	0.075	0.896	28.790	<0.001**	0.803
Aggression/Externalization Subscale	2.638	0.172	0.733	15.357	<0.001**	0.537
Misophonia Severity Scale	4.416	0.125	0.927	35.284	<0.001**	0.860

Dependent variable: Misophonia Total Score, B: Unstandardized Coefficient, SE: Standard Error, β: Standardized Coefficient (Beta), *t*: *t*-value, R(2): Coefficient of Determination (R squared), HIT-6: Headache Impact Test, MIDAS: Migraine Disability Assessment Test, DASS-21: Depression Anxiety Stress Scale-21, Y-BOCS-SR: Yale-Brown Obsession and Compulsion Scale-Self Reported Form, * *p* < 0.05, ** *p* < 0.01. Statistically significant values are indicated in bold.

## Discussion

Our study provides compelling evidence that misophonia is significantly more prevalent among individuals with migraine compared to healthy controls, with a 2.5-fold higher occurrence in the MG. Notably, migraine patients not only exhibited a higher prevalence of misophonia but also more severe symptoms and heightened emotional-behavioral reactions to trigger sounds. These findings underscore the clinical relevance of misophonia as a potential comorbidity in migraine, warranting further investigation into shared pathophysiological mechanisms and their implications for patient management.

In our cohort, over a quarter (25.8%) of migraine patients reported having aura. A striking finding was the higher prevalence of aura in MG-w-M (37%) compared to MG-wo-M (16.8%), suggesting a potential link between misophonia and cortical hyperexcitability in migraine with aura. Distinct sensory hypersensitivity profiles across migraine subtypes—particularly between those with and without aura—may influence how comorbid conditions like misophonia manifest and interact clinically (37). This association merits further exploration, as aura-related cortical spreading depression may interact with misophonia’s neural substrates, amplifying sensory and emotional dysregulation.

The MG with misophonia demonstrated significantly greater migraine-related disability, as evidenced by higher scores on the HIT-6 and MIDAS scales, compared to those without misophonia. Furthermore, 69.6% of migraineurs with misophonia reported moderate to severe disability due to migraine, compared to 58.7% of participants with migraine only. These findings indicate that the co-occurrence of misophonia and migraine is associated with increased migraine frequency and disability.

Previous studies have consistently demonstrated that sensory hypersensitivities—such as photophobia, phonophobia, osmophobia, and allodynia—are highly prevalent among individuals with migraine and have a significant clinical impact (38). These symptoms frequently co-occur and are thought to reflect either a higher disease burden or shared neurobiological mechanisms (39). Among these, photophobia and phonophobia are increasingly recognized as reliable markers of migraine severity and indicators of central sensitization (40, 41).

In our study, when we compared migraine patients with comorbid misophonia to those without, we observed a significantly higher prevalence of both interictal and ictal sensory hypersensitivities in the misophonia group. In contrast, no significant differences were found in interictal symptom prevalence between migraine patients with misophonia and controls with misophonia, suggesting that misophonia alone may not fully explain increased sensory sensitivity in the absence of migraine.

Importantly, ictal symptoms—particularly photophobia, phonophobia, osmophobia, and cutaneous allodynia—were consistently more prevalent and severe in MG-w-M, as reflected in ASC-12 scores. These findings support the hypothesis that misophonia may act as a sensory amplifier within the migraine phenotype, potentially exacerbating symptom severity through shared neurobiological pathways. In particular, the anterior insular cortex (AIC) and limbic system have been implicated in the processing of both auditory-emotional salience and pain-related sensory input (10, 42). The convergence of these networks may underlie the heightened sensory vulnerability observed in individuals with both migraine and misophonia. The heightened susceptibility to misophonia among migraine patients may be related to abnormal sensory processing mechanisms, which are well-documented in migraine pathophysiology (43–45). From a clinical standpoint, these results highlight the importance of screening for misophonia in migraine patients with prominent sensory complaints, as addressing this comorbidity may offer new avenues for symptom management and functional improvement.

The presence of misophonia may contribute to an increased psychiatric burden in individuals with migraine. This relationship aligns with prior research demonstrating elevated levels of anxiety, depression, and obsessive-compulsive traits among individuals with misophonia (46, 47). Our findings revealed that migraine patients with comorbid misophonia exhibited significantly higher psychiatric symptomatology compared to both healthy controls and migraine patients without misophonia, reinforcing earlier evidence linking misophonia with psychiatric conditions such as obsessive-compulsive disorder (OCD), depression, and anxiety (13, 18, 47, 48). Specifically, participants in the MG-w-M group showed markedly higher scores across all psychiatric scales, including nearly twice the level of stress (DASS-21) and an approximate three-point increase in depression and anxiety subscale scores.

These results suggest that misophonia may amplify sensory and emotional dysregulation within the migraine population. Considering that stress is a well-established trigger for migraine attacks (49), the heightened emotional reactivity induced by misophonic sensitivity to everyday sounds could potentially exacerbate migraine frequency and severity. The significant differences observed across multiple clinical and psychiatric measures (e.g., HIT-6, NRS, DASS-21, Y-BOCS-SR) support the view that misophonia is not merely a coincidental comorbidity but may meaningfully contribute to worse clinical outcomes in migraine. These findings underscore the importance of routinely screening for psychiatric symptoms in migraine patients with misophonia and incorporating targeted interventions into their care.

To further understand which variables contribute to misophonia severity among migraine patients, we performed univariate linear regression analyses. Our findings demonstrated that stress, OCD symptoms, allodynia, and migraine-related disability were significantly associated with misophonia total scores. Among psychiatric variables, stress had the strongest standardized coefficient (β = 0.516). Among migraine-specific variables, the MIDAS and HIT-6 scores showed significant predictive value. These results suggest that the severity of misophonia may be influenced by both sensory processing abnormalities and psychiatric comorbidities commonly seen in migraine.

The high R^2^ value for the Misophonia Severity Scale (0.860) and the Avoidance/Internalization subscale (0.803) highlights the importance of emotional and behavioral responses in misophonia symptomatology. These findings underscore the multifactorial nature of misophonia in the context of migraine and support the integration of psychological assessment into clinical evaluations. Behaviorally, MG-w-M participants were more likely to engage in avoidance behaviors (e.g., leaving environments) and reported violent thoughts in response to trigger sounds, reflecting the profound emotional toll of this comorbidity. These reactions may perpetuate auditory hypersensitivity and social withdrawal, further impairing quality of life. While no differences in sound-specific discomfort were observed between MG-w-M and misophonic controls, the heightened behavioral and emotional responses in migraine patients highlight the unique burden imposed by their dual diagnosis.

These clinical observations are consistent with the neurobiological model proposed by Kumar et al., who demonstrated that misophonia involves heightened activation of the AIC—a key hub of the salience network responsible for interoception and emotional regulation—in response to trigger sounds (10). Their findings suggest that misophonia reflects a dysfunction in assigning emotional salience to innocuous auditory stimuli, mediated by abnormal AIC connectivity with emotion-processing regions such as the amygdala and ventromedial prefrontal cortex. Importantly, the anterior insula has also been implicated in migraine pathophysiology. As reviewed by Borsook et al., the insula functions as a cortical hub integrating sensory, autonomic, and emotional signals (42). Migraine is characterized by altered sensory processing and interoceptive awareness, and repeated attacks can lead to functional and structural changes within the insula. In particular, both anterior and posterior insular regions show dynamic alterations related to pain intensity, affective processing, and even frequency of migraine episodes.

Neuroimaging studies also demonstrate altered limbic system activity in both migraine and misophonia, supporting the hypothesis of a shared affective-sensory processing dysfunction (50). Together, these findings from Kumar and Borsook suggest that dysfunction of the insula may serve as a shared neural substrate underlying both misophonia and migraine (10, 42). Our results support this view, as patients with higher misophonia severity also demonstrated increased stress, allodynia, and OCD symptoms—all of which may relate to hyperactivity within the insula and its connected circuits. This shared neural mechanism highlights the importance of evaluating insula-mediated affective and sensory processes when investigating comorbid conditions such as misophonia in migraine populations.

### Strengths and limitations

Our study is the first to systematically investigate misophonia in a well-characterized cohort of migraine patients, with diagnoses confirmed by headache specialists. The use of validated assessment tools and face-to-face clinical interviews enhances the accuracy and reliability of the collected data, especially when compared to online or self-reported surveys.

However, several limitations should be acknowledged. The control group was selected from hospital staff and relatives of patients, which introduces potential selection bias. Hospital staff may differ from the general population in terms of health literacy and stress exposure, while relatives may share genetic or environmental factors that influence migraine risk. While comparability in age and sex was established, unmeasured confounding factors may still affect the observed associations. The cross-sectional design also limits our ability to draw causal inferences.

In addition, the MQ used in the study does not distinguish misophonia from related conditions such as hyperacusis or phonophobia, which may lead to overlap in symptom reporting. This study was conducted in a tertiary headache center, where a high proportion of patients are typically on prophylactic treatment. Approximately two-thirds of our migraine patients were receiving preventive therapy; however, there was no significant difference in the use of prophylactic treatment between those with and without misophonia. Nevertheless, the potential influence of preventive therapies on psychiatric or sensory outcomes cannot be entirely ruled out, as these variables were not specifically adjusted for in our analysis. Effect sizes were not calculated, which restricts interpretation of the clinical relevance of our findings. Furthermore, the limited sample size prevented us from conducting more complex analyses, such as independent component analysis, to explore latent constructs.

These limitations highlight the need for future studies to use longitudinal designs, community-based control groups, multivariate statistical methods, and objective neurophysiological measures to better understand the relationship between migraine and misophonia and to refine diagnostic approaches.

### Clinical implications

The 2021 European consensus on migraine management emphasizes the importance of addressing comorbidities to improve outcomes (51). Our findings suggest that misophonia is an underrecognized comorbidity that may exacerbate migraine disability. Routine screening for misophonia in migraine patients could identify individuals at risk for greater sensory and psychiatric burden, enabling tailored interventions (e.g., cognitive-behavioral therapy, sound desensitization, or stress management). Multidisciplinary approaches targeting shared mechanisms (e.g., insula modulation) may offer novel therapeutic avenues.

## Conclusion

This study identifies misophonia as a highly prevalent and clinically significant comorbidity in migraine, linked to greater sensory hypersensitivity, psychiatric burden, and disability. Our results are in line with recent evidence indicating that disruptions in sensory and emotional brain networks may contribute to the development of complex comorbidities in headache disorders (10, 50, 52). By bridging a critical gap in the understanding of migraine comorbidities, this research lays the groundwork for future investigations into longitudinal trajectories, neurobiological underpinnings, and targeted interventions for misophonia in migraine patients.

## Data Availability

The datasets generated and analyzed during the current study are available from the corresponding author on reasonable request.
