# Supplementary material for: Assessing misophonia as a potential comorbidity in migraine patients compared to controls: a cross-sectional study
Source: Front Neurol. 2025 Jul 16;16:1545520. doi: 10.3389/fneur.2025.1545520 (PMC12308847; doi:10.3389/fneur.2025.1545520)
Supplement: Supplementary file 1 [file Data_Sheet_1.docx]

**SOCIODEMOGRAPHIC DATA FORM**

Participant Information

- Protocol Number: ________________________

- Date: ________________________

- Your Initials: ________________________

- Gender: ☐ Male ☐ Female ☐ Non-binary or gender diverse ☐ Prefer not to disclose

- Date of Birth**: ________________________

Educational Level:

- Literate / Primary School

- High School

- University

Smoking Habits:

- Non-Smoker

- Smoker (includes both active and passive smoking)

Alcohol Consumption Frequency:

- Does Not Consume

- Consumes (specify frequency: Monthly 1-3 times / Weekly 1-3 times / Daily)

Physical Activity Frequency:

- None

- 1-3 Times per Month

- 1-3 Times per Week

- Daily

Medical History (Check all that apply):

1. Depression

2. Anxiety

3. Fibromyalgia

4. Temporomandibular Joint Sensitivity

5. Restless Legs Syndrome

6. Sleep Disorder / Sleep Apnea

7. Hyperthyroidism / Hypothyroidism

8. Hypertension

9. Diabetes Mellitus

10. Heart Failure

11. Hypercholesterolemia

12. Stroke

13. Other Headaches

14. Gastritis / Ulcer

15. Other: _______

Family History of Migraine:

- Yes

- No

Medications Used Regularly:

- Please list any medications you take regularly: ________________________
