# Supplementary material for: Assessing misophonia as a potential comorbidity in migraine patients compared to controls: a cross-sectional study
Source: Front Neurol. 2025 Jul 16;16:1545520. doi: 10.3389/fneur.2025.1545520 (PMC12308847; doi:10.3389/fneur.2025.1545520)
Supplement: Supplementary file 2 [file Data_Sheet_2.docx]

**Clinical Features Data Form**

Participant Information

- Protocol Number: ________________________

- Date: ________________________

- Your Initials: ________________________

- Gender: ☐ Male ☐ Female ☐ Non-binary or gender diverse ☐ Prefer not to disclose

- Date of Birth**: ________________________

- Migraine Type

a) With Aura

b) Without Aura

c) Chronic

- Age of Onset:

At what age did you first experience migraine headache?

- Migraine Duration:

How many years have you been experiencing migraine headache?

- Migraine Frequency in the Last Month:

How many days in the past month did you experience a headache?

- Migraine Attack Frequency in the Past 3 Months:

a) Infrequent Episodic Migraine (0 to 3 days per month)

b) Frequent Episodic Migraine (4 to 14 days per month)

c) Chronic Migraine (15 or more days per month)

Medication Overuse Headache

- Yes / No

Photophobia

Does photophobia accompany the migraine?

a) Ictal Photophobia (During the migraine attack):

(0 - None, 1 - Mild, 2 - Moderate, 3 - Severe)

b) Interictal Photophobia (Between attacks):

(0 - None, 1 - Mild, 2 - Moderate, 3 - Severe)

Phonophobia

Does phonophobia accompany the migraine?

a) Ictal Phonophobia (During the migraine attack):

(0 - None, 1 - Mild, 2 - Moderate, 3 - Severe)

b) Interictal Phonophobia (Between attacks):

(0 - None, 1 - Mild, 2 - Moderate, 3 - Severe)

Osmophobia

Does osmophobia accompany the migraine?

a) Ictal Osmophobia (During the migraine attack):

(0 - None, 1 - Mild, 2 - Moderate, 3 - Severe)

b) Interictal Osmophobia (Between attacks):

(0 - None, 1 - Mild, 2 - Moderate, 3 - Severe)

Current Preventive Treatments

Which of the following preventive treatments are you currently using? (Select all that apply)

0. None

1. Antidepressants (e.g., TCA, SSRI, SNRI)

2. Anti-hypertensives (e.g., β-blockers, Ca channel blockers, ACE inhibitors)

3. Anti-epileptics (e.g., Topiramate, Valproate, Lamotrigine)

4. Botulinum Toxin A

5. Anti-CGRP Monoclonal Antibodies

6. Greater Occipital Nerve (GON) Block

7. Herbal Remedies

8. Other (Please specify)

Past Preventive Treatments

Which of the following preventive treatments have you used in the past? (Select all that apply)

0. None

1. Antidepressants (e.g., TCA, SSRI, SNRI)

2. Anti-hypertensives (e.g., β-blockers, Ca channel blockers, ACE inhibitors)

3. Anti-epileptics (e.g., Topiramate, Valproate, Lamotrigine)

4. Botulinum Toxin A

5. Anti-CGRP Monoclonal Antibodies

6. Greater Occipital Nerve (GON) Block

7. Herbal Remedies

8. Other (Please specify)
